# Prevalence of Anti-neural Autoantibodies in a Psychiatric Patient Cohort-Paradigmatic Application of Criteria for Autoimmune-Based Psychiatric Syndromes

**DOI:** 10.3389/fpsyt.2022.864769

**Published:** 2022-05-30

**Authors:** Niels Hansen, Aaron Levin Juhl, Insa Maria Grenzer, Kristin Rentzsch, Jens Wiltfang, Dirk Fitzner

**Affiliations:** ^1^Department of Psychiatry and Psychotherapy, University of Göttingen, Göttingen, Germany; ^2^Translational Psychoneuroscience, University of Göttingen, Göttingen, Germany; ^3^Euroimmun Reference Laboratory, Lübeck, Germany; ^4^German Center for Neurodegenerative Diseases (DZNE), Göttingen, Germany; ^5^Department of Medical Sciences, Neurosciences and Signaling Group, Institute of Biomedicine (iBiMED), University of Aveiro, Aveiro, Portugal; ^6^Department of Neurology, University Medical Center Göttingen, Göttingen, Germany

**Keywords:** delirium, psychiatry, neuronal autoantibodies, dementia, blood

## Abstract

**Background:**

Anti-neural autoantibodies associated with psychiatric syndromes is an increasing phenomenon in psychiatry. Our investigation aimed to assess the frequency and type of neural autoantibodies associated with distinct psychiatric syndromes in a mixed cohort of psychiatric patients.

**Methods:**

We recruited 167 patients retrospectively from the Department of Psychiatry and Psychotherapy, University Medical Center Göttingen for this study. Clinical features including the assessment of psychopathology *via* the Manual for Assessment and Documentation of Psychopathology in Psychiatry (AMDP), neurological examination, cerebrospinal fluid (CSF), magnetic resonance imaging (MRI) and electroencephalography (EEG) analysis were done in patients. Serum and or CSF anti- neural autoantibodies were measured in all patients for differential diagnostic reasons.

**Results:**

We divided patients in three different groups: (1) psychiatric patients with CSF and/or serum autoantibodies [PSYCH-AB+, *n* = 25 (14.9%)], (2) psychiatric patients with CSF autoantibodies [PSYCH-AB CSF+, *n* = 13 (7.8%)] and (3) those psychiatric patients without autoantibodies in serum and/or CSF [PSYCH-AB-, *n* = 131]. The prevalence of serum neural autoantibodies was 14.9% (PSYCH-AB+), whereas 7.2% had CSF autoantibodies (PSYCH-AB CSF+) in our psychiatric cohort. The most prevalent psychiatric diagnoses were neurocognitive disorders (61–67%) and mood disorders (25–36%) in the patients presenting neural autoantibodies (PSYCH-AB+ and PSYCH-AB CSF+). However, psychiatric diagnoses, neurological deficits, and laboratory results from CSF, EEG or MRI did not differ between the three groups. To evaluate the relevance of neural autoantibody findings, we applied recent criteria for possible, probable, or definitive autoimmune based psychiatric syndromes in an paradigmatic patient with delirium and in the PSYCH-AB+ cohort. Applying criteria for any autoimmune-based psychiatric syndromes, we detected a probable autoimmune-based psychiatric syndrome in 13 of 167 patients (7.8%) and a definitive autoimmune-based psychiatric syndrome in 11 of 167 patients (6.6%).

**Conclusions:**

Neural autoantibodies were detected mainly in patients presenting neurocognitive and mood disorders in our psychiatric cohort. The phenotypical appearance of psychiatric syndromes in conjunction with neural autoantibodies did not differ from those without neural autoantibodies. More research is therefore warranted to optimize biomarker research to help clinicians differentiate patients with potential neural autoantibodies when a rapid clinical response is required as in delirium states.

## Introduction

The detection of anti-neural autoantibodies in psychiatric patients is an increasing phenomenon in psychiatry (1–5). The reasons for this are not fully understood, although there is some evidence that might explain the phenomenon. The search for autoantibodies is advancing, the spectrum of potentially detectable autoantibodies has expanded, and several criteria have been developed (1) to better characterize these patient groups. It is important to understand whether neural autoantibodies rely on a potential autoimmune process. Knowing that information is especially important, as many cases of autoantibody-associated psychiatric syndromes reveal no obvious signs of an underlying encephalitis. In a review (1) we recently developed criteria for how to handle such patients, and we discuss the relevance of autoimmune processes as an origin for their phenotypic appearance. Delirium is especially interesting as neural autoantibodies have been reported so far only in a few studies in association with delirium (6). Delirium is a frequent condition in intensive care units and psychiatry wards with substantial mortality if no adequate treatment is provided. The causes are heterogeneous (7) and can comprise inflammation as a primary condition. Less is known about autoimmune processes in delirium, although we know that several cytokines related to autoinflammation can be elevated such as interleukin 6, interleukin-1ß and tumor necrosis factor alpha (8–10). Furthermore, cell membrane-surface (11–16) and intracellular antibodies (17, 18) have been reported in delirium states. Neural autoantibodies occur in cognitive impairment (1, 3–5), which is known to be a risk factor for triggering delirium (19). It thus makes sense to look for autoantibodies especially in patients with delirium and preexisting cognitive dysfunction. Our study aims to investigate the prevalence of neural autoantibodies in a psychiatry cohort. Furthermore, we will apply criteria for autoimmune-based psychiatric syndromes (1) in an index patient and in the whole cohort *via* detected neural autoantibodies to evaluate the autoimmune basis of psychopathology in these patients. Third, we will investigate whether delirium is frequent phenomenon in our psychiatric patient cohort, and estimate the neural autoantibody frequency in this subgroup of our psychiatric patient cohort.

## Methods

In this retrospective, observatory study, we enrolled 167 patients from our Department of Psychiatry and Psychotherapy, University Medical Center Göttingen between 2017 and 2020 in whom neural autoantibodies had been determined due to differential diagnosis. We formed two main psychiatric patient groups: (1) psychiatric patients with proof of serum and/or CSF autoantibodies (*n* = 25) and (2) psychiatric patients without proof of serum and/or CSF autoantibodies (*n* = 131). Eleven patients were excluded from analysis due to borderline autoantibody proof in serum or CSF. From the autoantibody-positive group we selected further those patients who demonstrated CSF autoantibody positivity (*n* = 12, Table 1). As mentioned above, we excluded patients with borderline proof of autoantibodies. Delirium was defined as a disorder of fluctuating attention and/or consciousness developing within a hours or days, coinciding with other disorders involving cognitive functions such as memory, orientation, language use, and not attributable to any other disease according to the Diagnostic and Statistical Manual of Mental Disorders fifth edition (DSM V) (20). Our cohort's neural autoantibodies were assessed in the Euroimmun laboratory in Lübeck by immunohistochemically analyzing serum and CSF probes *via* immunblots with Euroline and indirect immunofluorescent testing against neural antigens (Table 1). We sought autoantibodies against intracellular and cell-surface membrane antigens such as amphiphysin, α-amino-3-hydroxy-5-methyl-4-isoxazolepropionic acid receptors 1/2 (AMPAR1/2), Aquaporin 4, contactin associated protein 2 (CASPR2), CV2, dipeptidyl-peptidase-like 6 protein (DPPX), gamma aminobutyric acid B1/2 receptor (GABAB1/2R), glutamic acid decarboxylase (GAD65), HuD, Leucin Rich Glioma Inactivated Protein 1 (LGI1), Ma1/ Ma2, myelin oligodendrocytic protein (MOG), N-methyl-D-aspartate receptor (NMDAR), neurochondrin (NC), Ri, SOX1, TR, Yo and Zic4. Patient files were retrospectively assessed by two independent raters (ALJ, IMG) regarding psychopathology, clinical findings, and diagnostic data. We evaluated psychopathology by scoring applying a dichotomous design (symptom present = 1, symptom not present = 0). The Manual for Assessment and Documentation of Psychopathology in Psychiatry (AMDP) (21) was utilized for psychopathology symptoms. The AMDP's terminology comprises disorders of consciousness, orientation anomalies, impaired memory and attention, formal thought disorders, compulsions and worries, delusions, disorders of perception, ego disturbances, disturbances of affect, impaired drive and psychomotor activity, circadian disturbances and other problems such as suicidal behavior, self-harm or social withdrawal. If any symptom associated with each of the aforementioned subordinate terms was present, that category was considered impaired. We applied the International Classification of Psychiatric Disorders ICD10 (International classification of diseases, 10th edition) to classify our psychiatric cohort's psychiatric disorders. The CSF was measured and analyzed in the Neurochemistry Laboratory in the Department of Neurology, University Medical Center Göttingen. Markers of neurodegeneration such as ß-amyloid 42 (Aß42), ß-amyloid 40 (Aß40), total tau protein, phosphorylated tau protein 181 (ptau181) were determined in the same laboratory. If any of the following items was present in a patient–namely, an actual or past tumor, movement disorder, early adverse response to antidepressant or antipsychotic drugs, severe cognitive dysfunction, altered consciousness, seizures, optic hallucinations, infectious prodrome or aphasia, mutism or dysarthria, we evaluated the positivity factor as a relevant autoimmune indicator according to our recent recommendations (1). This study was conducted according to the current version of the Declaration of Helsinki, and approved by our local Ethics Committee. The statistical approach we took to compare two groups with different conditions was Fisher's exact test. A *p*-level of *p* <0.05 was regarded as significant.

**Table 1:** Demographic and clinical data of psychiatric patients associated with anti-neural autoantibodies.

**No**.	**Psychiatric diagnosis**	**Neurological diagnosis**	**Antibody serum**	**Antibody CSF**	**Cutt off**	**Test method for antibody detection**
1	F31.2	-	Ma2 +	Ma2 +	< (+)	Immunblot (Euroline)
2	F06.2	G04.8,	CV2/CRMP5++	CV2/CRMP5(+)	< (+)	Immunblot (Euroline)
3	F02.2	G04.8	NMDAR +	-	<1:10	IgM IFT
4	F05.0, F06.7	-	NMDAR ++ (1:32)	-	<1:10	anti-neural IgG IFT
5	F00.1	-	NC +++ (1:3200)	NC+++ (1:32)	<1:100 (serum)	anti-neural IgG IFT
			Titin ++	Titin ++	<1:10 (CSF)	Immunblot (Euroline)
6	F33.1	G04.8	-	Yo+	< (+)	Immunblot (Euroline)
7	F00.0	G30.0	Glycine + (1:100)	-	<1:10	anti-neural IgG IFT
8	F06.7	-	Yo +	na	< (+)	Immunblot (Euroline)
			DNER +	na	< (+)	Immunblot (Euroline)
			GAD65+	na	< (+)	Immunblot (Euroline)
9	F42.2, F33.1, F41.0, F51.0	-	REC +	na	< (+)	Immunblot (Euroline)
10	F02.8, F32.2	G04.8	KCNA2 ++ (1:100)	-	<1:10	anti-neural IgG IFT
11	F06.7	G40.3, G23.3	Titin +	-	< (+)	Immunblot (Euroline)
			MOG++ (1:100)	-	<1:10	anti-neural IgG IFT
12	F00.2	-	REC +	na	< (+)	Immunblot (Euroline)
13	F33.2, F13.1	-	NMDAR ++	NMDAR + 1:1	<1:10	anti-neural IgG IFT
14	F05.1	-	Zic4 +	na	< (+)	Immunblot (Euroline)
15	F06.7	-	NMDAR ++ (1:100)	-	<1:10	anti-neural IgG IFT
16	F33.2	-	Yo +	-	< (+)	Immunblot (Euroline)
17	F06.7	-	Glycin ++ (1:32)	-	<1:10	anti-neural IgG IFT
18	F00.1	-	KCNA2 ++ (1:32)	-	<1:10	anti-KCNA2 IgG IFT
19	F20.0	-	NMDAR ++ (1:100)	-	<1:10	Anti-NMDAR IgG IFT
20	F06.2	G04.8	NMDAR+++ (1:1000)	NMDAR+++(1:100)	<1:10	Anti-NMDAR IgG IFT
21	F06.2	G04.8	CASPR2 ++++ (1:10000)	CASPR2 ++++ (1:3200)	<1:10	Anti-CASPR2 IgG IFT
22	F02.8	-	NMDAR ++ (1:100)	-	<1:10	Anti-NMDAR IgG IFT
23	F00.2	G45	Titin +	Titin+	< (+)	Immunblot (Euroline)
24	F00.1, F33.0	-	Neuropil ++ (1:320)	Neuropil (1: 3.2)	<1:10	Anti-neural IgG IFT
25	F06.7, F51.0	-	IgLON5++++ (1:3200)	IgLON5++++ (1:320)	<1:10	Anti.IgLON5 IgG

*CASPR2, Contactin-associated protein-like 2-antibody; CSF, cerebrospinal fluid; CV2/CRMP5, cronveinten 2/ collapsin response mediator protein-5; GAD65, glutamic acid decarboxylase 65; IFT, immunofluorescence testing; IgG, immunoglobulin G; IgM, immunoglobulin M; KCNA2, potassium voltage-gated channel subfamily A member 2; MOG, myelin oligodendrocyte glycoprotein; NC, neurochondrin; NMDAR, N-methyl-D-aspartate-receptor; N0., number; REC, recoverin*.

## Results

### Psychiatric Patient Cohort

We screened 167 patients originating from our inpatient and outpatient unit in the Department of Psychiatry and Psychotherapy, University Medical Center Göttingen for the occurrence of neural autoantibodies. Thirty-six of the 167 (22%) psychiatric patients presented autoantibodies including

borderline autoantibody positivity. After excluding patients with borderline autoantibody positivity, we yielded a group of 25 autoantibody-positive psychiatric patients (PSYCH-AB+, Table 1) from our cohort of 167 psychiatric patients (14.9%). Our PSYCH-AB+ presented serum Ma2, CV2/CRMP5, Yo, Myelin, NMDAR, NC, Titin, Glycin, REC, DNER, KCNA2, MOG, Zic4, CASPR2, SOX1, Hu, IgLON5 antibodies (Table 1). Furthermore, from our PSYCH-AB+ group we identified a further group showing CSF autoantibodies (PSYCH-AB+ CSF, *n* = 12, 7.2%). Taking the number of CSF analyses (*n* = 159) in our patients into account, we detected CSF neural autoantibodies in 12/159 (7.5%). The autoantibody spectrum of PSYCH-AB+ CSF comprised Ma2, CV2/CRMP5, NC, Titin, Yo, MOG, CASPR2, Zic4, SOX1, Ma, Hu, REC and IgLON5 (Table 1). We detected relevant autoimmune indicators in 24 of 25 (96%) of our PSYCH-AB+ patients, and in all patients with psychiatric symptoms and CSF autoantibodies. No differences emerged between groups in clinical autoimmune indicators. Age and gender did not differ between groups (Table 2). PSYCH-AB+ as well as PSYCH-AB+ CSF patients did not differ in their psychiatric diagnosis according to ICD10 compared to PSYCH-AB- patients (Table 2). Figure 1A illustrates the spectrum of autoantibodies in PSYCH-AB+ patients in those patients with an F00-F09 diagnosis, whereas Figure 1B shows the spectrum of neural autoantibodies in PSYCH-AB+ patients with F30-F39 diagnoses as the main diagnoses. Figure 1C represents the spectrum of autoantibodies in PSYCH-AB+CSF patients with F00-F09 diagnosis, whereas Figure 1D shows the spectrum of autoantibodies in in PSYCH-AB+CSF patients with F30-F39 diagnosis. The most prevalent psychiatric diagnoses were neurocognitive disorders (61–67%) and mood disorders (25–36%) in our patients presenting neural autoantibodies (PSYCH-AB+ and PSYCH-AB CSF+). The psychopathology did not differ between groups regarding orientation, attention and memory disturbances, worries and compulsions, delusions, hallucinations, ego disturbances, circadian disturbances, suicidality or self-harm. However, mood dysfunction, psychomotor abnormalities or a thought disorder were much more present in PSYCH-AB- than in PSYCH-AB+ patients. Furthermore, psychomotor disturbances were much more frequent in PSYCH-AB CSF+ than in PSYCH-AB+ patients. Other clinical and laboratory parameters regarding neurological deficits, tumors, CSF parameters, MRI and EEG results did not differ between PSYCH-AB+ or PSYCH-AB+ CSF vs. PSYCH-AB- groups (Table 2).

**Table 2:** Demographic, clinical and laboratory data of groups.

	**Psychiatric patients with AB+**		**PSYCH-AB-**
	**1. PSYCH-AB+ CSF**	**2. PSYCH-AB+**	
**Number (% of cohort)**	12/167 (7.2%)	25 /167 (14.9%)	131/ 167 (78.7%)
**Age**	60.6 ± 17.8	61.9 ± 16.4	55.7 ± 18.1
**Gender (female)**	5/12 (42%)	9/25 (36%)	65/131 (49.8%)
**Diagnosis groups**			
F00-09	8/12 (67%)	16/25 (64%)	56/131 (42.9%)
F10-19	1/12 (8.3%)	0/25 (0%)	3/131(2.3%)
F20-29	0/12 (0%)	1/25 (4%)	24/131 (18.5%)
F30-39	3/12 (25%)	7/ 25 (36%)	37/131 (28.2%)
F40-49	0/12 (0%)	2/ 25 (8%)	7/131 (5.3%)
F50-59	1/12 (8.3%)	0/ 25 (0%)	0/131 (0%)
**Psychopathology**			
Orientation disturbances	7/12 (58%)	13/ 25 (52%)	44/123 (35.8%)
Attention and memory disturbances	9/12 (75%)	18/ 25 (72%)	102/123 (83.3%)
Formal thought disorder	10/12 (83%)	12/ 25 (48%)*	87/123 (71%)*
Worries and compulsions	4/12 (33%)	6/ 25 (24%)	29/ 123 (23.8%)
Delusions	3/12 (25%)	3/ 25 (12%)	25/123 (20.3%)
Hallucinations	1/12 (8.3%)	2 / 25 (8%)	15/123 (12.2%)
Ego disturbances	1/12 (8.3%)	0/ 25 (0%)	0/ 123 (0%)
Mood disturbances	8/12 (67%)	13/ 25 (52%)*	99/123 (80.6%)*
Psychomotor disturbances	8/12 (67%)^#^	6/ 25 (24%)*^#^	74/123 (60.2%)*
Circadian disturbances	2/12 (17%)	3/ 25 (12%)	23/123 (18.8%)
Suicidality	0/12 (0%)	1/ 25 (4%)	18/123 (14.7%)
Self-harm	0/12 (0%)	2/25 (8%)	7/123 (5.7%)
**Neurological deficits**			
Cerebral nerve palsy	2/12 (17%)	2/23 (8.7%)	7/114 (6.1%)
Oculomotor palsy	2/12 (17%)	1/23 (4.3%)	3/114 (2.6%)
Hypomimia face	1/12 (8.3%)	1/23 (4.3%)	5/114 (4.4%)
Hypoesthesia face	1/12 (8.3%)	0/23 (0%)	2/114 (1.8%)
Hyperesthesia face	0/12 (0%)	1/23 (4.3%)	1/114 (0.87%)
Paresis upper extremities	0/12 (0%)	0/23 (0%)	2/114 (1.8%)
Paresis lower extremities	0/12 (0%)	0/23 (0%)	5/114 (4.4%)
Hyporeflexia	0/12 (0%)	2/23(8.7%)	14/114 (12.3%)
Hyperreflexia	0/12 (0%)	0/23 (0%)	7/114 (6.25%)
Rigor	0/12 (0%)	0/23 (0%)	11/114 (9.2%)
Spasticity	0/12 (0%)	0/23 (0%)	3/114 (2.6%)
Tremor	1/12 (8.3%)	1/23 (4.3%)	15/114 (13.2%)
Loss of movement	1/12 (8.3%)	3/23 (13.1%)	22/114 (19.6%)
Hypoesthesia upper extremities	0/12 (0%)	1/23 (4.3%)	1/114 (0.87%)
Hypoesthesia lower extremities	2/12 (16%)	5/23 (21.7%)	14/114 (12.3%)
Thermhypesthesia lower extremities	0/12 (0%)	0/23 (0%)	1/114 (0.87%)
Paresthesia extremities	0/12 (0%)	1/23 (4.3%)	8/114 (7.01%)
Ataxia	1/12 (8.3%)	4/23 (13.1%)	14/114 (12.3%)
Postural instability	1/12 (8.3%)	6/23 (26%)	26/114 (22.8%)
Gait disturbance	2/12 (16%)	6/23 (26%)	25/114 (22%)
**Clinical autoimmune indicator**			
Actual or recent tumor	2/12 (16%)	1/25 (4%)	24/ 128 (19%)
Movement disorder	0/12 (0%)	5/24 (21%)	25/ 118 (21%)
Adverse response to AP or AD	1/12 (8.3%)	4/25 (16%)	29/ 122 (24%)
Severe cognitive dysfunction	11/12 (92%)	22/25 (88%)	86/ 129 (67%)
Altered consciousness	0/11 (0%)	0/24 (0%)	0/129 (0%)
Seizures	3/11 (27%)	2/24 (8.3%)	6/134 (4.5%)
Optic hallucinations	0/11 (0%)	1/24 (24%)	10/123 (8%)
Infectious prodrome with fever	0/11 (0%)	0/23 (0%)	1/124 (0.8%)
Aphasia, mutism, dysathria	4/12 (33%)	8/24 (33%)	30/121 (24.8%)
**CSF**			
Cell count (<5 μg/L)	8 ± 2.2	1.4 ± 2.4	1.5 ± 7.1
Lymphocytes in %	59.4 ± 40.6	64.6 ± 41	58.5 ± 39.8
Monocytes in %	10.1 ± 8.9	12.1 ± 9.4	14.5 ± 14.3
Total protein count (mg/L)	544 ± 220	481 ± 252	416 ± 193
Albumin (mg/L)	348 ± 134	307 ± 160	278 ± 143
IgG (mg/L)	51 ± 30	44.6 ± 30.2	33.7 ± 22.5
IgA (mg/L)	10.7 ± 5.2	4.6 ± 3.3	4.00 ± 4.11
IgM (mg/L)	1.2 ± 0.9	0.94 ± 0.83	1.06 ± 3.,94
Quotient CSF/ serum albumin %	9.4 ± 5.8	7.8 ± 5.2	6.6 ± 3.5
Quotient CSF/serum IgG %	4.6 ± 2.2	3.9 ± 2.4	3.4 ± 2.2
Quotient CSF/serum IgA %	2.4 ± 1.3	2.0 ± 1.3	1.7 ± 1.16
Quotient CSF/serum IgM %	1.2 ± 0.7	0.9 ± 0.67	0.76 ± 0.76
Intrathecal IgG synthesis	1/13 (7.7%)	3/21 (14.3%)	11/124 (8.8%)
Blood brain barrier disturbance	4/13 (30.7%)	4/21 (19%)	25/124 (20.2%)
**MRI**			
Generalized atrophy	2/11 (18%)	7/19 (37%)	39/115 (34.5%)
Focal atrophy	3/11 (17%)	7/19 (37%)	27/115 (23.8%)
Hippocampal atrophy	0/11 (0%)	1/19 (5.2%)	6/115 (5%)
Vascular lesions	5/11 (45%)	9/19 (47%)	43/115 (37%)
Extended cerebrospinal fluid rooms	2/11 (18%)	8/19 (43%)	28/115 (24.4%)
**EEG**			
Temporal focal slowing	5/10 (50%)	8/13 (61.5%)	30/74 (40.1%)
Temporal epileptic potentials	0/10 (0%)	1/13 (7.7%)	3/74 (4.1%)
Non-temporal focal slowing	5/10 (50%)	7/13 (54%)	28/74 (38.4%)
Non-temporal epileptic potentials	0/10 (0%)	0/13 (0%)	2/74 (2.7%)
**Tumor**	2/10 (20%)	8/25 (32%)	27/167 (16%)

*CSF, cerebrospinal fluid; IgA, immunoglobulin A; IgG, immunoglobulin G; IgM, immunoglobulin M.*

*Statistics:*

* *p <0.05 Fisher's exact test: PSYCH-AB CSF+ vs. PSYCH-AB− patients.*

# *p <0.05 Fisher's exact test: PSYCH-AB CSF+ vs. PSYCH-AB− patients*.

**Figure 1:**
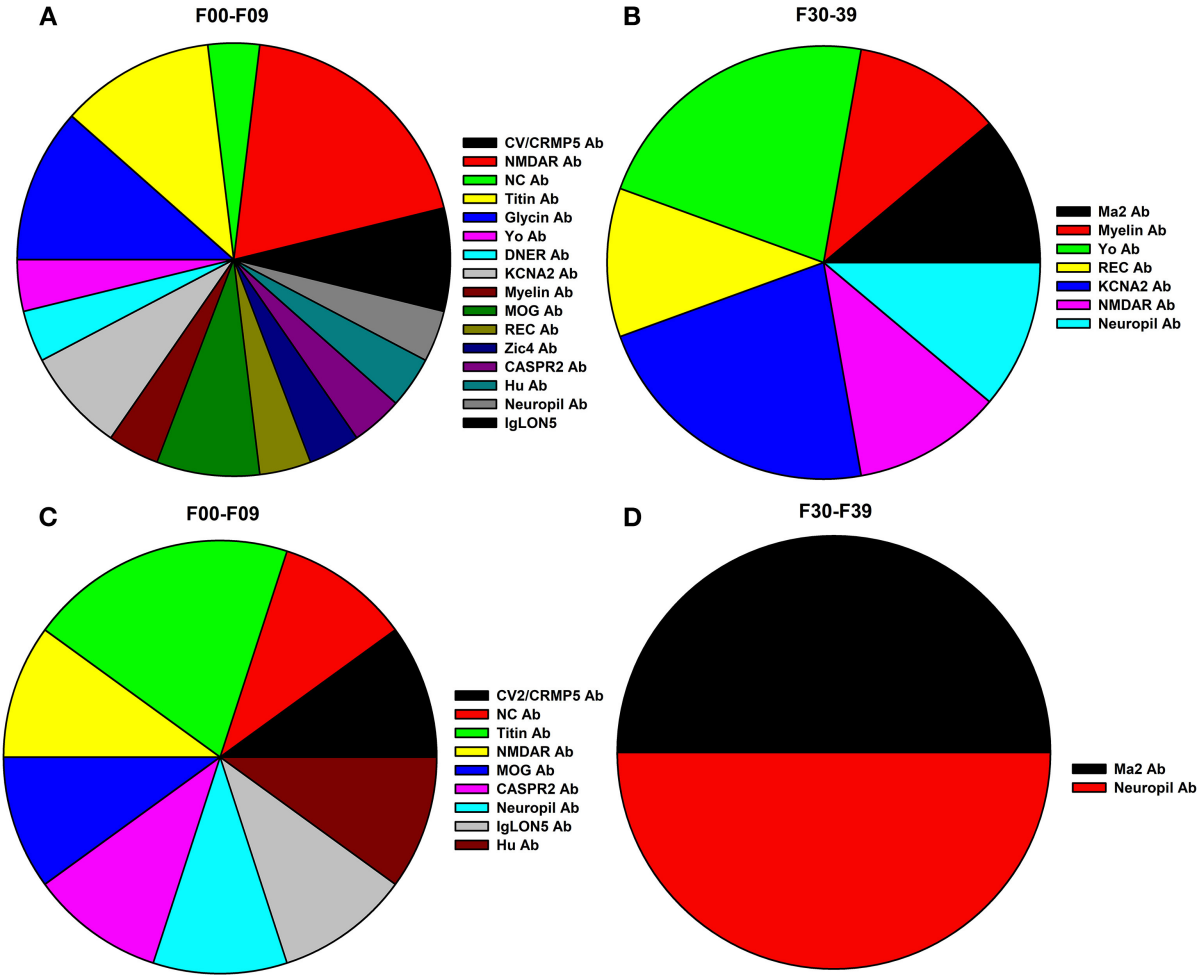
Circle diagrams of anti-neural autoantibody spectrum in psychiatric patients. The autoantibody spectrum of PSYCH-AB+ patients with F00-09 diagnoses are shown in **(A)**, F30-F39 diagnoses are illustrated in **(B)** (PSYCH-AB+ patients). In **(C)** the spectrum of autoantibodies of PSYCH-AB CSF+ is shown for F00-F09 according to ICD 10 and in **(D)** (PSYCH-AB CSF+) the diversity of autoantibodies associated with ICD10 F30-39 are depicted. Ab, autoantibodies; CASPR2, Contactin-associated protein 2; CV2/CRMP5, cronveinten 2/ collapsin response mediator protein 5; DNER, delta/notch-Like EGF Repeat Containing; MOG, myelin oligodendrocyte glycoprotein; NMDAR, N-methyl-D-aspartate receptor; NC, neurochondrin; PSYCH-AB CSF+, psychiatric patients with CSF autoantibodies; PSYCH-AB+, psychiatric patients with CSF and/or serum autoantibodies; REC, recoverin.

### Delirium Patients From Psychiatric Patient Cohort

We diagnosed a delirium in 5 of these 167 (3%) psychiatric patients. Two of those five revealed neural autoantibodies (40%). One presented cell-surface autoantibodies against NMDAR, and the other patient against the intracellular antigen target Zic4. Both showed autoantibodies against intracellular and membrane-surface targets. A psychorganic syndrome was present in all patients with delirium. The most often reported psychopathological features were orientation deficits and memory disturbances in 100%, mood disturbances in 80%, formal thought disorder in 60%, and psychomotor disturbances in 40% (Table 3). Two patients with delirium were diagnosed with additional psychiatric syndromes according to AMDP, i.e., a paranoid-hallucinatory and neurologic syndrome in patient #1, and an apathic syndrome in patient #5. Interestingly, both patients with neural autoantibodies and delirium had a history of a tumor as a relevant autoimmune indicator, whereas no tumor history was present in the three patients with delirium but no neural autoantibodies. No axonal or amyloid-based neurodegeneration was detected in one delirium patient with NMDAR antibodies, as his normal values were below the cut-off levels for tau protein, tau 181, Aß42, Aß40, the Aß42/40 ratio. Four of five delirium patients had suffered dementia before their delirium occurred. We had no CSF from one patient with dementia and delirium coinciding with neural autoantibodies. A neurodegenerative dementia is highly likely in 2/ 3 patients, as they presented elevated levels of total tau protein and ptau 181. We identified elevated tau protein and total tau protein in all patients with delirium (Table 3). MRIs were abnormal in 2–3 of five patients, and focal or generalized atrophy was diagnosed in 40% and vascular lesions in 60% of delirium patients (Table 3). Furthermore, EEGs revealed temporal and non-temporal slowing in 50% of patients (Table 3).

**Table 3:** Demographic, clinical and laboratory data of five delirium patients.

**Age**	**72.8 ± 4.8**
**Gender**	**2 females**
**Psychopathology**	
**Orientation disturbances**	**5/5 (100%)**
**Attention and memory disturbances**	**5/5 (100%)**
**Formal thought disorder**	**3/5 (60%)**
**Worries and compulsions**	**0/5 (0%)**
**Delusions**	**0/5 (0%)**
**Hallucinations**	**2/5 (40%)**
**Ego disturbances**	**0/5 (0%)**
**Mood disturbances**	**4/5 (80%)**
**Psychomotor disturbances**	**2/5 (40%)**
**Suicidality**	**0/5 (0%)**
**Self-harm**	**0/5 (0%)**
**CSF**	
**Cell count /μl (<5 μg/l)**	**1.25 ± 0.7**
**Lymphocytes in %**	**73.8 ± 34.5**
**Monocytes in %**	**24.8 ± 14.4**
**Total protein count mg/L**	**473 ± 278**
**Albumin mg/ L**	**270 ± 132**
**IgG mg/ L**	**37.1 ± 18.3**
**IgA mg/ L**	**4.9 ± 2.6**
**IgM mg/ L**	**0.7 ± 0.51**
**Quotient CSF/ serum albumin %**	**7.2 ± 3.7**
**Quotient CSF/serum IgG %**	**3.5 ± 1.9**
**Quotient CSF/serum IgA %**	**2.2 ± 1.3**
**Quotient CSF/serum IgM %**	**0.74 ± 0.53**
**Intrathecal IgG synthesis**	**0/5 (0%)**
**Blood brain barrier disturbance**	**0/5 (0%)**
**t-tau pg/ml (<450 pg/ml)**	**750 ± 528**
**ptau181 pg/ml (<61 pg/ml)**	**79.2 ± 41**
**Aß42 pg/ml (>450 pg/ml)**	**893 ± 461**
**Aß40**	**10,946 ± 5,210**
**Ratio Aß42/Aß40 × 10 (>0.5)**	**0.83 ± 0.41**
**MRI**	
**Generalized atrophy**	**2/5 (40%)**
**Focal atrophy**	**2/5 (40%)**
**Hippocampal atrophy**	**1/5 (20%)**
**Vascular lesions**	**3/5 (60%)**
**Extended cerebrospinal fluid rooms**	**3/5 (60%)**
**EEG**	
**Temporal focal slowing**	**2/4 (50%)**
**Temporal epileptic potentials**	**0/4 (0%)**
**Non-temporal focal slowing**	**2/4 (50%)**
**Non-temporal epileptic potentials**	**0/4 (0%)**
**Tumor**	**2/5 (40%)**

*CSF, cerebrospinal fluid; IgG, immunoglobulin G; IgA, immunoglobulin A; IgM, immunoglobulin M; t-tau, total tau protein; ptau181, phosphorylated tau protein 181; Aß42, amyloid beta 42; Aß40, amyloid beta 40; ratio Aß42/Aß40, ratio amyloid beta 42/ amyloid beta 40*.

### Applying the Criteria of an Autoimmune-Based Psychiatric Syndrome

#### Paradigmatic Application in a Patient With Delirium

To better understand the application of our recently developed criteria (1), we applied them paradigmatically in a patient with delirium presenting serum NMDAR autoantibodies. This patient has a minor neurocognitive disorder that warranted further consideration of an autoimmune basis [see for relevant diagnoses (1)]. He has presented optic hallucinations, a tumor, a movement disorder and early treatment resistance. Each of these clinical features would fulfill one required criterion for diagnosing a potentially autoimmune-based psychiatric syndrome. A possible autoimmune-based psychiatric syndrome can therefore be suspected. Furthermore, these four factors coincide with laboratory findings such as focal temporal slowing in EEG and the presence of serum NMDAR autoantibodies. We therefore assumed a probable autoimmune-based delirium in our patient. We ruled out a definitive autoimmune-based delirium, as this patient presented no IgG CSF NMDAR autoantibodies. The other patient with delirium also had a probable autoimmune-based psychiatric syndrome.

#### Application in the Cohort of Patients With Neural Autoantibodies

Our applying of the aforementioned criteria for autoimmune-based psychiatric syndromes in our PSYCH-AB+ patients yielded 13 patients with a probable and 11 with a definitive autoimmune-based psychiatric syndrome. We thus identified a 13/167 (7.8%) prevalence of probable autoimmune-based psychiatric syndromes and an 11/167 (6.6%) prevalence of definitive autoimmune-based psychiatric syndromes in our cohort.

## Discussion

Our main finding is that autoantibodies against intracellular and membrane-surface antigens are present in 15% patients from a psychiatric cohort suffering from delirium and cognitive deficits. Most patients in our cohort revealed a neurocognitive or mood disorder and neural autoantibodies, although their phenotypic appearance did not different from those without neural autoantibodies. 7.8% of patients presented a probable and 6.6% a definitive autoimmune-based psychiatric syndrome. These results show that autoimmunity seems to occur in a relevant fraction of psychiatric patients who also suffer from a cognitive or mood dysfunction. Although not the main focus of our investigation, we observed 2 of 5 delirium patients with neural autoantibodies probably originating from autoimmune processes. If replicated in larger patient samples with delirium, this finding might indicate that delirium is present in a relevant subgroup that apparently contains a moderate proportion of patients with neural autoantibodies. The main clinical features of the PSYCH-AB+ patients in our psychiatric cohort were cognitive impairment, mood disturbances, formal thought disorder, psychotic symptoms and psychomotor abnormalities. Formal thought disorder, mood disturbances, and psychomotor abnormalities were much more often present in PSYCH-AB- than in PSYCH-AB+ patients, indicating a potentially less affective and less formal thought disorder dysfunctional psychopathology-profile pattern in PSYCH-AB+ patients. However, because of our heterogeneous group of patients with different diagnoses and our study's retrospective character, we cannot draw any clear conclusions, as more homogeneous patient cohorts need to be examined. Another important fact to consider in our delirium patients is dementia, as dementia in delirium is a frequent phenomenon, displays a neuropsychological profile different from that in patients without dementia, and it predisposes to depression states (19). A recent meta-analysis highlighted the clinical relevance of prior dementia for delirium, as its prevalence is high (48.9%) and accompanied by worse functional and cognitive dysfunction in the long-run (22). Thus, in our delirium patients, it is unclear if the autoantibodies present are associated with the dementia condition or the delirium, and more research is necessary to clarify this question. Neural autoantibody- associated dementia (1, 3–5) is a growing field in immunopsychiatry that deserves further attention. In an earlier study we showed that a substantial proportion of patients with cognitive impairment reveal an association with neural autoantibodies (4). As our patient sample of delirium patients was too small, no clear conclusions can be drawn about the frequency of neural autoantibodies in delirium. It is nevertheless obvious that neural autoantibody-associated delirium occurs, and that it and is not infrequent in patients with delirium in psychiatric cohorts already suffering from cognitive impairment. These findings support the urgency of further large-scale studies to investigate the frequency of autoantibody associations in a large cohort of delirium patients, especially in those with and without prior cognitive impairment. We detected NMDAR and Zic4 antibodies in the serum of delirium patients suggesting possible CNS inflammation. However, these patients delivered no other evidence supporting an CNS inflammation, as it is not characterized by pleocytosis or relevant neurodegeneration. The abnormalities in our cohort's brain MRIs were unspecific, i.e., there was no general brain atrophy or cerebral microangiopathy-more evidence that our patients with delirium and NMDAR and Zic4 autoantibodies present no relevant brain damage. However, as the number of our patients with neural autoantibodies is too small, with no conclusions are possible about whether neural autoantibodies in delirium can cause relevant brain damage. Brain damage associated with delirium might originate from different pathological mechanisms such as inflammation-triggered neurodegeneration. Neurodegeneration in delirium states is frequent, and results from a preexisting neurodegenerative disorder or from novel neurodegeneration caused by various mechanisms [see (7) for review]. The treatment of autoantibody-associated delirium is another topic deserving more research in large-scale studies, especially considering that immunotherapy for autoantibody-associated delirium is not always mandatory. The justification for taking an immunotherapeutic approach in this scenario comes from therapeutic success in autoantibody-associated psychiatric syndromes (23) and autoimmune encephalitis (24, 25). In particular, studies should be done in those patients with delirium and autoantibodies who do fulfill autoimmune-encephalitis criteria or who present no clear signs of brain damage associated with delirium.

If a patient presents many autoimmune indicators, we strongly recommend taking a blood sample to assess neural autoantibodies, although more investigation is needed before therapeutic guidelines can be formulated. Furthermore, neural autoantibodies should be determined in those patients who suffer a delirium and have a history of cancer. Cancer is often diagnosed in delirium patients (26), and it might play a role, as cancer immunity lowers the threshold for inducing delirium. However, it is difficult to differentiate between patients in whom the surgical procedure itself leads to an inflammation triggering delirium, and those delirium patients in whom pre-existing cancer immunity causes delirium (not the surgical procedure and related inflammatory conditions). Paraneoplastic autoantibodies are good indicators of underlying cancer immunity. Our second patient with autoantibodies presented the paraneoplastic Zic4 antibody, supporting his potential for cancer-related immunity. Morbidity and mortality are high in patients with cancer and delirium (27), and delirium is particularly frequent in patients suffering from advanced malignancies (28). Risk factors in cancer patients for developing delirium include (besides the aforementioned paraneoplastic neuropsychiatric syndromes), primary CNS tumors or secondary CNS tumors entailing brain metastasis or meningeal metastasis, or encephalopathy following brain radiation for certain tumor types (29).

## Limitations

The limitations of this study concern the small sample size of delirium patients and the heterogeneous spectrum of neural autoantibodies detected that does not enable us to draw conclusions about autoantibody patient subgroups. In addition, the incidence or prevalence of autoantibody-associated delirium can only be estimated in large trials that are being planned. The strength of our study is that our results demonstrate that psychiatric symptoms are associated with a variety of neural autoantibodies with autoimmune indicators pointing toward a possible autoimmune origin, although their significance remains unclear. Further large-scale prospective studies combined with the assessment of biomarkers targeting brain damage and inflammation will have to be performed to better characterize the role of neural autoantibodies. Another study limitation is the application of criteria of autoimmune-based psychiatric syndromes from a single research group (1) only representing the concept of suggested criteria, and is not proof of an autoimmune basis of the psychiatric syndrome itself. Thus, a straightforward approach in a future investigation would be to apply several differential classification criteria and compare their relevance regarding the potential causal autoimmunity from various molecular parameters in blood and CSF, neural autoantibodies, and psychiatric syndromes. We suggest utilizing as different classifications the criteria for autoimmune encephalitis (30) as recently applied in a cohort of psychiatric patients diagnosed with psychiatric autoimmune encephalitis (24) or autoimmune psychosis (31), to compare the value of assessing a psychiatric syndrome's suspected autoimmune basis.

## Conclusions

Our retrospective study describes a 15% prevalence of neural autoantibodies in a mixed psychiatric cohort of patients suffering from with mainly neurocognitive and mood disorders. However, detailed analysis revealed that a much lower percentage had an autoimmune origin of symptomatology (7.8% probable, 6.6% definitive autoimmune basis). Further research is needed to draw attention to this field, as potential autoimmune involvement in psychiatric disorders has been neglected so far. Biomarker research will be crucial to improving the diagnostics and therapy of such patients, especially when rapid clinical diagnostics and therapies are required, as when they suffer delirium states.

## Data Availability Statement

The raw data supporting the conclusions of this article will be made available by the corresponding author, without undue reservation.

## Ethics Statement

The studies involving human participants were reviewed and approved by Ethics Committee of the University Medical Center Göttingen. Written informed consent for participation was not required for this study in accordance with the national legislation and the institutional requirements. Written informed consent was obtained from the individual(s) for the publication of any potentially identifiable images or data included in this article.

## Author Contributions

NH wrote the manuscript. AJ and IG performed data collection. KR did part of the laboratory testing. JW and DF revised the manuscript for important intellectual content. All authors contributed to the article and approved the submitted version.

## Funding

Funding is derived from the Open Access Fund of the University of Göttingen. JW is supported by an Ilídio Pinho professorship, iBiMED (UIDB/04501/2020) at the University of Aveiro, Portugal.

## Conflict of Interest

The authors declare that the research was conducted in the absence of any commercial or financial relationships that could be construed as a potential conflict of interest.

## Publisher's Note

All claims expressed in this article are solely those of the authors and do not necessarily represent those of their affiliated organizations, or those of the publisher, the editors and the reviewers. Any product that may be evaluated in this article, or claim that may be made by its manufacturer, is not guaranteed or endorsed by the publisher.
